# Does resilience mediate the association between mental health symptoms and linguistic markers of trauma processing? Analyzing the narratives of women survivors of intimate partner violence

**DOI:** 10.3389/fpsyg.2023.1211022

**Published:** 2023-06-13

**Authors:** Marco Castiglioni, Cristina Liviana Caldiroli, Gian Mauro Manzoni, Rossella Procaccia

**Affiliations:** ^1^Department of Human Sciences “R. Massa, ” University of Milano-Bicocca, Milan, Italy; ^2^Faculty of Psychology, eCampus University, Novedrate, Italy

**Keywords:** depression, PTSD, intimate partner violence (IPV), resilience, sense-making, trauma processing

## Abstract

Intimate partner violence (IPV) is a serious issue for women from all cultures and backgrounds. Studies on the negative consequences of violence suggest that women with a history of abuse are more likely to display depressive and PTSD symptoms. However, recent research has focused on the mechanisms underpinning resilience and the processing of traumatic memories, including linguistic markers and how they may reflect the mental health of traumatized individuals. In this study, we analyzed trauma narratives to investigate whether resilience mediates the impact of PTSD and depression symptoms on five trauma-processing mechanisms (cognitive processing, emotional processing, perceived threat to life, self-perspective, and integration of traumatic memories). Forty-three abused women (mean age = 38.74 years; *SD* = 9.41) wrote about their traumatic experiences and completed instruments assessing their levels of PTSD, depression, and resilience. We used LIWC software to analyze the women's narratives in terms of linguistic markers of psychological processing. Mediation analysis indicated that resilience fully mediated the impact of mental health symptoms on emotional processing, perceived threat to life, and integration of traumatic memories and partially mediated cognitive processing and self-perspective. We discuss the clinical implications of these findings, emphasizing the need to focus on the resources and strengths of women survivors of abuse in designing targeted psychological interventions.

## 1. Introduction

### 1.1. Intimate partner violence: definition, prevalence, and psychological sequelae

Intimate partner violence (IPV) is a major social and health problem that may be defined as “*behavior within an intimate relationship that causes physical, sexual or psychological harm, including acts of physical aggression, sexual coercion, psychological abuse, and controlling behaviors”* (World Health Organization (WHO), 2010, p. 11). IPV may be perpetrated by current or former partners of either sex, although most offenders are men (Holmes et al., 2007). IPV is driven by the offender's need for power and involves coercion and intimidation as well as violence (White and Satyen, 2015). International data suggest that almost one-third of all women have experienced some form of IPV (Galovski et al., 2021). In Italy, an estimated 2,800,000 women (between the ages of 16 and 70 years) have endured one or more episodes of sexual or physical violence by a partner or cohabitant (Troisi, 2018). During the COVID-19 health emergency, IPV became an even more critical problem (Barbara et al., 2022), given the combined impact of enforced isolation in the home with abusive partners, exacerbated financial worries, and loss of employment.

IPV is associated with negative physical and mental health outcomes over the short and long term. Victims of IPV are at increased risk of physical illness and behavioral and mental problems including substance use, social impairment, insomnia, anxiety, mood disorders, and suicidality (Campbell, 2002; Kramer et al., 2004; Burke et al., 2005; Woods, 2005). Depression and post-traumatic stress disorder (PTSD) are also frequently associated with IPV (Golding, 1999; Bauer et al., 2000; Beydoun et al., 2012; Kim and Lee, 2013). In an early meta-analysis, Golding (1999) investigated the prevalence of mental health problems among women with a history of IPV, finding that 31% to 84.4% met the criteria for PTSD, 47.6% met the criteria for depression, and 17.9% met the criteria for suicidality. More recently, a meta-analysis by Beydoun et al. (2012) suggested that IPV was moderate to strongly associated with depression. Specifically, compared to non-victimized peers, female victims of IPV were 2–3 times more likely to develop major depression, and 1.5–2 times as likely to suffer from elevated depressive symptoms and postpartum depression. Similarly, Kim and Lee (2013) found that depression was more prevalent in women with a history of IPV; they also identified a bi-directional relationship between IPV and depression, with women who had been diagnosed with depression twice as likely to have suffered IPV than the general population. Finally, victims of IPV are at increased risk of PTSD and comorbidity between depression, PTSD, and other mental and physical disorders (Iverson et al., 2011; Lagdon et al., 2014).

### 1.2. Resilience and linguistic markers of trauma processing

Up to the 1980s, disease models drove problem-focused studies designed to investigate maladjustment following adverse events. However, the last 20 years have seen a shift to resilience models, which focus on individuals' ability to build up their strengths. Resilience is the combination of factors that helps a person to resume functioning following a damaging event. Curtis and Cicchetti (2003) proposed that resilience is at play when an individual displays competent functioning despite having undergone significant adversity. According to Luthar et al. (2000), resilience entails positive adaptation in the face of adverse experiences. Rutter (2006) suggested that resilience is the outcome of protective processes that do not eliminate risk or adversity but enable the individual to cope more effectively with them. Other authors have similarly argued that resilience cannot be reduced to a single isolated dimension but rather is shaped by multiple protective factors at the individual and environmental levels (Werner and Smith, 2001; Walsh, 2015). Resilience has been studied in relation to adverse events including chronic disease (Kim et al., 2019), child abuse and maltreatment (Di Blasio et al., 2005; Procaccia, 2011; Marriott et al., 2014), the COVID-19 public health emergency (Castiglioni and Gaj, 2020; Procaccia et al., 2021; Castiglioni et al., 2023a,b; Negri et al., 2023), and IPV (Bradley et al., 2005; Anderson et al., 2012; Nasution et al., 2020; Crann and Barata, 2021; Fernández-Álvarez et al., 2022).

Many trauma studies have adopted a qualitative approach, inferring subjects' processing of traumatic or stressful events from the analysis of their narratives (Pennebaker and Chung, 2007; Procaccia et al., 2014, 2018, 2020). Language use has long been investigated within clinical and social research. Scholars have argued that people's verbal behavior conveys key information about their emotional, physical, and mental states (Junghaenel et al., 2008). Focusing on individuals' spontaneous choice of words, the authors have shown that the use of certain terms in writing is related to aspects of mental wellbeing and mental illness. The linguistic features of trauma survivors' accounts of their experience can offer more direct and “unfiltered” feedback about how they are processing and integrating traumatic memories that may be obtained from self-assessment or interview measures (Kleim et al., 2018). Furthermore, by narrating their traumatic experiences, individuals can free themselves of automatic and unwanted thoughts, begin to construe the meaning of their adverse experience, and enhance their emotion regulation, with consequent gains in resilience and general wellbeing (Pennebaker and Chung, 2007). While many different methods are used to analyze language use (Pennebaker et al., 2003), one widely adopted approach is to evaluate linguistic style in terms of the specific kinds of words that individuals use, independently of the context and the semantic content of their discourse (Pennebaker and King, 1999). The drawback of this method is the loss of key information that can only be inferred from the context. Its advantage, however, is that it facilitates mixed-method research designs yielding more generalizable outcomes. It frequently involves computerized language analysis, whereby the words in a text sample are counted and assigned to pre-defined word categories.

Researchers have investigated how narratives can reflect various underpinnings of psychological adjustment or maladjustment, such as cognitive and emotional processing, perceived threat to life, self-distanced/immersed perspectives, and integration of traumatic memories.

Cognitive models suggest that engaging in deeper *cognitive processing* during traumatic experiences is associated with a lower risk of PTSD. Conversely, individuals who primarily engage in surface-level processing, concentrating on the visual, perceptual, and sensory aspects of traumatic episodes, subsequently display more marked PTSD symptoms (Ehlers and Clark, 2000; Brewin, 2011). Greater deployment of cognitive terms such as “why,” “reason,” “realize,” and “understand,” implying “causal” and “insightful” thought processes, is related to greater physical and mental wellbeing. More frequent cognitive markers in a trauma narrative imply the greater ability to reflect on the causal features of the traumatic event, which, in turn, facilitates meaning-making processes (Pennebaker, 2000; Warner et al., 2006). In relation to *emotional processing*, some authors (Pennebaker and Francis, 1996; Frisina et al., 2004) found that greater expression of positive emotion (via terms such as “calm,” “joy,” and “happiness”) and moderate expression of negative emotion (via terms such as “angry” “guilt,” and “shame”) were associated with better adjustment. Conversely, Ozer et al. (2003) reported that more frequent expressions of negative emotion in the narratives of traumatized subjects were the strongest predictor of later PTSD. Next, *perceived threat to life* (reflected in the use of death-related terms), which arises from feeling helpless and believing one's mental and physical safety to be endangered during traumatic events, is also associated with PTSD (Alvarez-Conrad et al., 2001; Wilker et al., 2017).

Moving on to *self-distanced perspectives* (expressed via second- and third-person pronouns, singular or plural, such as “you,” “she,” “he,” and “they”), this mechanism enables individuals to observe themselves during negative episodes. It is associated with more effective processing of negative feelings and lower levels of distress because it helps the individual to see the “bigger picture” and thus to make sense of the traumatic experience. Conversely, *self-immersed perspectives* (expressed via first-person singular pronouns, such as “*I*,” “*me*,” and “*my*”) lead individuals to narrowly focus on the specific details of their experience, fostering rumination and depressive symptoms (Pronin and Ross, 2006; Kross and Ayduk, 2011).

Finally, the *integration of traumatic memories* into autobiographical memory is a mechanism that promotes mental health because it restores the sense of continuity and self-consistency undermined by the traumatic experience. The degree to which subjects have integrated their traumatic memories is reflected in the verb tenses used in their narratives (Pennebaker and Chung, 2011). Specifically, present and future tenses suggest more effective processing of traumatic events, greater resilience, greater ability to cope, and greater self-confidence in relation to the future. On the contrary, greater use of past tenses implies that the subject may be unable to move on from the traumatic event.

Focusing now on IPV, our previous study (Procaccia and Castiglioni, 2022) with 77 female abuse victims showed that emotional and cognitive processing mediated the impact of a three-session expressive writing intervention on participants' levels of PTSD and depression. Crann and Barata (2021) examined the narratives of women abuse survivors, finding that resilience was largely the outcome of cognitive, emotional, and behavioral change during the abusive relationship itself. In a qualitative study on IPV survivors, Nasution and colleagues (2020) concluded that the main key to resilience is the subjects' analysis of events, which significantly affects their ability to react: a positive mindset is associated with greater motivation to transform suffering into meaning.

Given this background, the present study aimed to investigate the processing of traumatic experience in the narratives of women IPV survivors, focusing on the role of resilience in mediating the effects of PTSD and depressive symptoms.

Specifically, we hypothesized that:

(H1) higher levels of PTSD and depression would be associated with poorer traumatic memory processing, as reflected in fewer cognitive terms, fewer positive emotion terms, more negative emotion terms, stronger perceived threats to life, weaker self-distanced perspectives, and stronger self-immersed perspectives, and poorer integration of memories (more frequent past-tense verbs and less frequent present- and future-tense verbs).
(H2) higher levels of resilience would be associated with better traumatic memory processing, as reflected in more cognitive terms, more positive emotion terms, fewer negative emotion terms, lesser perceived threat to life, stronger self-distanced perspectives, and weaker self-immersed perspectives, and enhanced integration of memories (more infrequent past-tense verbs and more frequent present- and future-tense verbs).
(H3) resilience would mediate the effects of PTSD and depressive symptoms on the linguistic markers of traumatic memory processing.

## 2. Materials and methods

### 2.1. Participants

For the purposes of the present study, we focused exclusively on the expressive writing group (Pennebaker and Francis,1996) in our previously reported research (Procaccia and Castiglioni, 2022). Participants were recruited through services for abused women settled in Northern and Central Italy. The inclusion criteria for the study were: (1) having been a victim of IPV; (2) being over 18; (3) being sufficiently proficient in written and spoken Italian; and (4) currently living in safe conditions (have been completely separated from the abusive partner for at least 30 days). The 43 women who joined the study (mean age = 38.74 years; *SD* = 9,41) were predominantly Italian, with a medium-high level of education, and were currently employed. Most had been married to or stably cohabiting with their partners; they were victims of chronic and multiple forms of abuse and the majority had been separated from the abusing partner for <6 months. Participants displayed severe PTSD symptoms (LASC cutoff values by King et al., 1995), and over half (58.1%) presented moderate to severe depression (based on the BDI-II cutoff values by Beck et al., 1996; Italian validation by Ghisi et al., 2006). Nevertheless, we also identified high levels of resilience (based on the RSA cutoff values recommended by Friborg et al., 2003; see Table 1). The data were collected over the period January–August 2022.

**Table 1:** Demographics.

**Total number**	**43**	
Age (years)		
Mean (SD)	38.74 (9.41)
Min–max	19	59
Nationality		
Italian	28	65.1%
Not italian	15	34.9%
Education		
Middle school license	11	25.6%
Degree	24	55.8%
Post-graduate degree	8	18.6%
Occupational status		
Employed	29	67.4%
Unemployed	14	32.6%
Marital status		
Married or cohabiting	31	72.10%
Stable partner	12	27.90%
Children		
Children	32	74.4%
No children	11	25.6%
Number of children		
Min–max	0	4
Mean (SD)	1.56 (3.22)	
<1 year	6	13.95%
>1 year	37	86.05%
Separation from abusing partner		
<6 months	27	62.80%
>6 months	16	37.20%
Type of victimization		
Psychological abuse	43	100%
Physical abuse	34	79.06%
Sexual abuse	28	65.11%
More than one type of abuse	36	83.72%
PTSD		
Mean (SD)	29.34 (13.22)
Min–max	4	50
Depression		
Minimal range	9	20.09%
Mild depression	9	20.09%
Moderate depression	9	20.09%
Severe depression	16	37.2%
Resilience	97.12 (32.15)
Min–max	50	129

### 2.2. Procedure

All participants completed a consent form that explained the aims of the study, a socio-demographic questionnaire, and the full battery of research instruments. The consent form highlighted the risks associated with the study, including distress from recalling traumatic experiences, and advised the participants that they were free to withdraw at any time. The women filled out the questionnaires individually at home. Next, they received instructions for a three-session writing task based on the standard expressive writing protocol proposed by Pennebaker and Francis (1996). Specifically, they were asked to write about their traumatic experiences with a focus on their deepest emotions and feelings, in their home, for 20 min a day, for three consecutive days. One week after finishing the writing assignment, they again completed the battery of research instruments. The study complied with the Ethics Code of the Italian Psychological Association and was approved by the Ethics Committee of eCampus University (n. 05/2021). Participants' personal information was handled in compliance with the General Data Protection Regulation (GDPR) and EU Regulation 2016/679.

### 2.3. Measures

*Demographic characteristics*: Participants were asked to state their age, ethnic background, level of education, number of children, marital/relationship status, number of years of victimization, and type of abuse (sexual abuse, physical abuse, and psychological abuse).

*Beck Depression Inventory* (Beck et al., 1996; Italian validation by Ghisi et al., 2006; BDI-II) was administered to investigate depressive symptoms. This 21-item tool assesses the cognitive, affective, motivational, and behavioral dimensions of depression. Each item is rated on a 4-point scale from 0 (never) to 3 (always), yielding a global depression score of 63 points max. Based on the Italian validation study, we adopted a cutoff score of >12 to establish whether depression was present. Scores from 13 to 19 indicate minimal depression; from 20 to 28, moderate depression; and from 29 to 63, severe depression. Cronbach's α coefficient has ranged from 0.80 to 0.87 in normative or clinical samples (Lee et al., 2017). In this study, the α coefficient was 0.85.

*Los Angeles Symptom Checklist* (LASC; King et al., 1995) was administered to assess PTSD symptoms. This 43-item self-report instrument measures global distress due to trauma exposure, the severity of overall PTSD, and the severity of individual PTSD symptoms (namely, re-experiencing, avoidance/numbing, and hyperarousal). Previous studies found high internal consistency, with α coefficients ranging from 0.88 to 0.95 (Foy et al., 1997). In this study, the α coefficient was 0.91. The LASC items were translated into Italian following a back translation procedure.

*Resilience Scale for Adults* (RSA, Friborg et al., 2003; Italian version by Capanna et al., 2015) was administered to assess resilience. This 33-item self-report scale measures adults' protective resilience factors. Each item is a 7-point semantic differential scale with a positive attribute at one end and the opposite negative attribute at the other end. To reduce acquiescence bias, half of the items are reverse scored. Higher scores indicate higher levels of protective resilience factors. The instrument displays a six-factor structure expressing individual–familial and social dimensions of resilience, namely, perception of self; planned future; social competence; structured style; family cohesion; and social resources. RSA has been shown to have a good internal consistency, with Cronbach's alpha values ranging from 0.79 to 0.88 for the overall scale, and from 0.67 (structured style) to 0.81 (self-perception) for the six subscales (Hjemdal et al., 2011). In our study, the α coefficient was 0.82.

### 2.4. Linguistic analysis

We transcribed the women's narratives verbatim and subjected them to a Linguistic Inquiry Word Count (LIWC) analysis (Pennebaker et al., 2001, Italian dictionary by Agosti and Rellini, 2007) to identify patterns and frequencies of language use. The LIWC program calculates the frequency with which words occur in a text. It recognizes ~2,000 words and codes them to a set of linguistic categories (such as pronouns, past, present, and future tense, negative and positive emotion terms, and insight words). It computes the total number of words in a text and the ratios of the different linguistic categories to the overall corpus.

In this study, we set out to evaluate the relationship between the participants' linguistic markers at the beginning of the writing task and the baseline levels of the other variables under study (as opposed to changes that came about as a result of the writing intervention). We thus limited our linguistic analysis to the first of the three written narratives produced, assessing the texts in relation to:

1. Cognitive processing, as reflected in the use of causal terms (e.g., “reason,” “because,” and “thus”) and insight terms (e.g., “realize,” “see,” and “understand”);
2. Emotional processing, as reflected in references to positive emotions (e.g., “happy,” “joy,” and “elated”) and negative emotions (e.g., “sad,” “mad,” “guilt,” and “angry”);
3. Perceived threat to life, as reflected in the use of death-related terms (e.g., “die,” “death,” “loss,” “grief,” “threat,” and “danger to life”);
4. Self-perspective, as reflected in markers of a self-immersed perspective (first-person singular pronouns such as “I,” “me,” “my,” and “mine”) vs. a self-distanced perspective (second- and third-person singular or plural pronouns such as “you,” “she,” “he,” “they,” “him,” “her,” and “them”);
5. Integration of traumatic memories, as reflected in the relative frequencies of past-, present-, and future-tense verbs.

### 2.5. Statistical analyses

The descriptive analysis entailed computing participants' baseline scores for PTSD, depression, and resilience, as well as for the above-listed linguistic markers. We also conducted Pearson's r correlational analyses to investigate the associations between PTSD, depression, resilience, and linguistic markers of traumatic memory processing. Next, to test our final hypothesis, we performed a set of hierarchical multiple regression analyses, in accordance with the mediational model proposed by Baron and Kenny (1986). All statistical analyses were conducted using SPSS 21.

## 3. Results

### 3.1. Associations between PTSD, depression, resilience, and linguistic markers of traumatic processing

The correlational analyses (see Table 2) showed that both PTSD and depression were negatively correlated with resilience. Furthermore, PTSD was positively correlated with a self-immersed perspective, perceived threat to life, and past-tense verb forms, but negatively correlated with cognitive processing, positive emotions, a self-distanced perspective, and present- and future-tense verb forms. Similarly, depression was negatively correlated with a self-distanced perspective, cognitive processing, positive emotions, and future-tense verb forms, but positively correlated with a self-immersed perspective and past-tense verb forms. Finally, resilience was negatively correlated with a self-immersed perspective, perceived threat to life, and past-tense verb forms, but positively correlated with cognitive processing, positive emotions, a self-distanced perspective, and future-tense verb forms.

**Table 2:** Correlational analysis.

	**Depression**	**PTSD**	**Resilience**	**Cognitive elaboration**	**Positive emotion**	**Negative emotion**	**Threat to life**	**Self-distance**	**Self-immersed**	**Pass tense**	**Present tense**	**Future tense**
1. Depression	1	0.794^**^	−0.571^**^	−0.403^**^	−0.370^*^	0.088	0.014	−0.307^*^	0.774^**^	0.857^**^	−0.428^**^	−0.600^**^
2. PTSD	0.794^**^	1	−0.561^**^	−0.553^**^	−0.325^*^	0.22	0.311^*^	−0.355^*^	0.520^**^	0.656^**^	−0.402^**^	−0.531^**^
3. Resilience	−0.571^**^	−0.561^**^	1	0.327^*^	0.305^*^	−0.028	−0.317^*^	0.394^*^	−0.412^**^	−0.517^**^	0.175	0.786^**^

** Sig ≤ 0.01.

* Sig ≤ 0.05.

### 3.2. Mediational models

**Figure 1:**
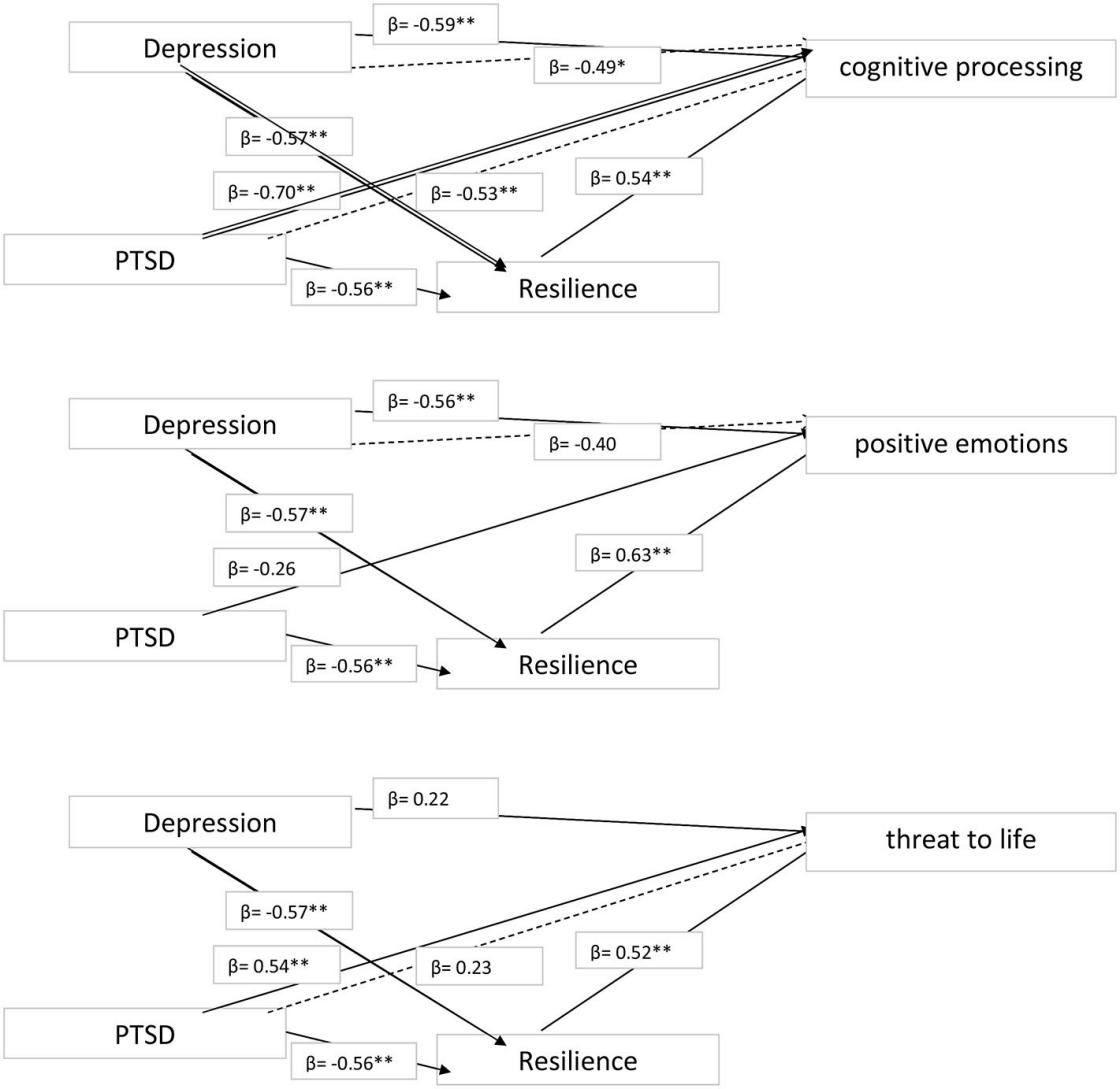
Mediation model for cognitive processing, positive emotion, and threat to life. ^*^p < 0.05, ^**^p < 0.01.

**Figure 2:**
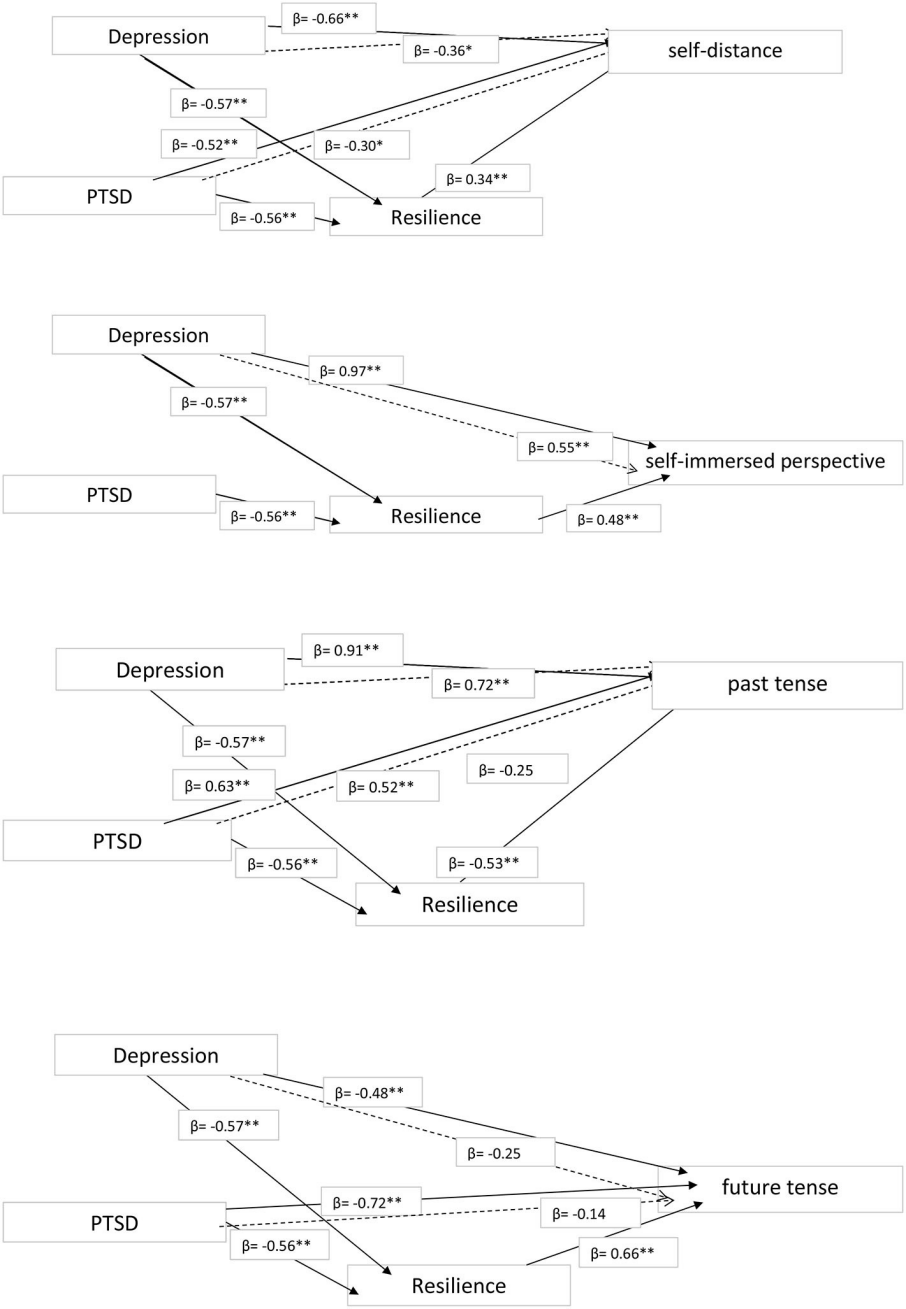
Mediation Model for self-distanced and self-immersed perspectives; past tense and future tense. ^*^p < 0.05, ^**^p < 0.01.

We performed multiple regression analyses, with the linguistic markers of traumatic processing as the dependent variables, depression and PTSD as predictors, and resilience as the mediator (see Figures 1, 2), finding that:

1. Poorer cognitive processing was predicted most strongly by PTSD (β = −0.70; *t* = −2.91, *p* = 0.006) followed by depression (β = −0.59; *t* = −2.21, *p* = 0.008). These effects were partially mediated by resilience.
2. Less frequent reporting of positive emotions was predicted by depression only (β = −0,56; *t* = −2.18, *p* = 0.009), and this effect was fully mediated by resilience. There were no significant effects for negative emotion.
3. Greater perceived threat to life was predicted by PTSD only (β = −0.54; *t* = −2.98, *p* = 0.007), and this effect was fully mediated by resilience.
4. A self-distanced perspective was predicted most strongly by lower levels of depression (β = −0.66; *t* = −6.20, *p* = 0.001), followed by lower levels of PTSD (β = −0.52; *t* = −2.78, *p* = 0.008), and these effects were partially mediated by resilience. A self-immersed perspective was predicted by higher levels of depression only (β = 0–97; *t* = 6.10, *p* = 0.001), and this effect was partially mediated by resilience.
5. Regarding the integration of traumatic memories, greater use of past-tense verb forms was predicted most strongly by depression (β = 0.91; *t* = 9.20, *p* = 0.001), followed by higher levels of PTSD (β = 0.63; *t* = 6.20, *p* = 0.002). These effects were partially mediated by resilience. Neither PTSD nor depression predicted the use of present-tense verb forms. Finally, the use of future-tense verbs was predicted most strongly by lower levels of PTSD (β = −0.72; *t* = −7.19, *p* = 0.0001), followed by lower levels of depression (β = −0.48; *t* = −2.33, *p* = 0.02), with both effects fully mediated by resilience.

## 4. Discussion

Consistently with past studies (Pico-Alfonso et al., 2006; Ellsberg et al., 2008), we found that women survivors of IPV display severe depressive and PTSD symptoms, but also strong resilience. Previous research on IPV has mainly been focused on its negative psychological and physical consequences, yet a growing body of positive psychology research has also examined resilience and how women cope with and move through their history of abuse (Nasution et al., 2020; Crann and Barata, 2021; Fernández-Álvarez et al., 2022).

Numerous studies have investigated mental health by analyzing the personal narratives of groups of subjects, including IPV survivors (Pennebaker and Chung, 2007; Kleim et al., 2018). Our results show once again that linguistic markers reflect abused women's mental health status, and that PTSD and depression negatively influence the processing of traumatic experiences. Nevertheless, we also found that resilience consistently mediates these relationships, mitigating distress and promoting the processing of traumatic memories. More specifically, our findings confirm that cognitive processing and emotional processing are key to overcoming trauma. The narratives of women with more severe PTSD contain fewer causal and insight terms, reflecting the difficulty in attributing meaning and coherence to traumatic experiences. Women who are more depressed, on the other hand, make fewer references to positive emotions in their accounts. However, resilience buffers against the impact of mental distress, fostering positive feelings, counteracting negative brooding, and promoting meaning-making by stimulating causal inferences and insights. Following the cognitive processing theory, we suggest that engaging in cognitive and emotional processing of trauma can foster reflection about its meaning, thus enhancing subjects' sense of coherence and psychological wellbeing (Pennebaker and Seagal, 1999). Both cognitive and emotional processing are amplified by resilience, reducing the negative emotional valence of traumatic memories and facilitating their integration into autobiographical memory. More specifically, intrusive memories are experienced less frequently, while enhanced emotion regulation means that even stressful thoughts and memories cause less arousal (Davidson et al., 2002). This makes it easier for female survivors to reorganise and make sense of their traumatic memories and consequently to regain a sense of continuity and coherence.

Similarly, our data confirm that PTSD predicts a greater preoccupation with death, reflecting the perceived threat to their physical and psychological integrity that trauma has induced in IPV victims. Nevertheless, greater resilience buffers women against such feelings of fragmentation and powerlessness, enabling them to cope better with trauma.

Our results further show that the negative consequences of IPV, especially depression, predict the adoption of a self-immersed perspective. Specifically, women with more severe depressive symptoms make greater use of first-person singular pronouns and less use of third-person pronouns in their narratives (Junghaenel et al., 2008). Following previous research on self-control and psychological distance (Fujita et al., 2006), we may plausibly assume that a self-immersed perspective will predispose women to focus narrowly on recounting the concrete details of their experience rather than on reconstruing them in ways that provide insight and closure of the traumatic events (Kross and Ayduk 2011). Self-distancing is one of the psychological mechanisms that allow individuals to reflect on negative experiences in an adaptive way, and to attribute adversity with meaning so that it can cease to be a constant source of mental suffering. Hence, resilience fulfills a “buffering” function because it partially mediates the effects of PTSD and depression, favoring the processes of self-distancing and meaning-making that ultimately mitigate distress.

Finally, we found that resilience mediates the effect of depression and PTSD on the integration of traumatic memories. Specifically, women with more severe mental distress displayed a greater focus on the past than on the present and future, whereas resilience was associated with a more future-oriented perspective. This outcome aligns with previous findings that resilience and the capacity to overcome trauma are associated with a greater ability to move on from the past and to plan for the future with a sense of self-efficacy (Pennebaker and Chung, 2011).

While our results are promising, some methodological limitations of this study should be taken into account. First, the small sample size precludes the generalization of the findings. Second, the statistical analysis did not include some of the variables that could potentially impact mental health symptoms (such as duration and severity of the abuse) and, consequently, also affect processes of resilience. Third, the women completed the writing task unsupervised in their own homes and, therefore, it was not possible to record their reactions during the task or to keep intervening variables under control.

Additionally, writing interventions by definition are mainly of use to those with a medium to high level of education, meaning that less well-educated individuals may not benefit as much. Furthermore, we drew on an individualized model of resilience in this study, but it would be interesting to engage with a more ecological and context-sensitive framework. Finally, we inferred psychological mechanisms implicated in trauma processing based on a relatively small number of decontextualized words, which cannot capture the full complexity of the underlying processes.

Nevertheless, our study points up the key role of resilience in the processing of traumatic events. It also reaffirms the importance of investigating language as a mirror of mental health, and the value of automated text analysis, which should prompt broader research programs with clinical populations. The study of spontaneous language can inform targeted therapeutic interventions, specifically designed to promote resilience and thus counteract the negative effects of violence. Future research could usefully explore the relationship between the various dimensions of resilience (perception of self; planned future; social competence; structured style; family cohesion; and social resources) and the different processes at play in women's narratives. In addition, it would be enlightening to explore not only how depression and PTSD affect women's narrative processes, but also the inverse, that is to say, how the way women construct their narratives affects their mental state. The insights gained by pursuing these follow-up lines of inquiry should lead to further improvements in clinical practice.

## Data availability statement

The raw data supporting the conclusions of this article will be made available by the authors, without undue reservation.

## Ethics statement

The studies involving human participants were reviewed and approved by Ethic Committee of eCampus University. The patients/participants provided their written informed consent to participate in this study.

## Author contributions

All authors listed have made a substantial, direct, and intellectual contribution to the work and approved it for publication.
